# An Exploratory Analysis of Transcriptional Responses to Peanut Exposure in *Drosophila melanogaster*

**DOI:** 10.3390/ijms27104545

**Published:** 2026-05-19

**Authors:** Carlos Hernandez, Alexis M. Hobbs, Joseph J. Dolence, Peng Xiao, Kimberly A. Carlson

**Affiliations:** 1 Department of Biology, University of Nebraska at Kearney, Kearney, NE 68849, USA; hernandezc3@lopers.unk.edu (C.H.); hobbsa2@unk.edu (A.M.H.); dolencejj@unk.edu (J.J.D.); 2 Department of Genetics, Cell Biology and Anatomy, University of Nebraska Medical Center, Omaha, NE 68198, USA; peng.xiao@unmc.edu

**Keywords:** peanut, *Drosophila melanogaster*, qRT-PCR, next-generation sequencing

## Abstract

Much remains to be learned about how the innate immune system responds following exposure to food allergens, such as peanut. *Drosophila melanogaster* is an untapped model system for examining this topic because of its conserved innate immune pathways, although it lacks adaptive immunity. The objective of this study was to determine if innate immune-regulated genes within the *D. melanogaster* genome were transcriptionally regulated by exposure to peanut. RNA samples were analyzed by qRT-PCR and next-generation sequencing. qRT-PCR data shows a significant downregulation of *Dorsal* and *Relish* at day 24. Next-generation sequencing data identified a limited number of differentially expressed genes at days 15 and 30, including those involved in structural, metabolic, and digestive functions. Taken together, our data suggests modest and limited transcriptional changes associated with peanut exposure. This study provides an initial framework for investigating how food allergens, such as peanut, likely influence innate immune-associated gene expression.

## 1. Introduction

Food allergies represent a growing global health challenge, with peanut allergy among the most severe and persistent forms of food hypersensitivity. In the United States, peanut allergies affect nearly six million children and adults and remain a leading cause of fatal food-induced anaphylaxis [1]. The prevalence of food allergies appears to be increasing in recent decades. However, evidence that supports an increased frequency remains incomplete due to the high cost and potential risks of conducting oral food challenges in large populations; instead, most rely on self-reporting or parental reporting [2]. Studies that do utilize oral food challenges estimate prevalence between 5 and 10% in Westernized regions, with peanut being among the most common allergens in older children (>5 years) [3]. This increasing prevalence could reflect multiple converging factors, including changes in diet, specifically outdated guidance to avoid peanuts in infancy, environmental exposures, and immune dysregulation. To date, the underlying mechanisms of allergic sensitization remain incompletely understood [4]. Current clinical approaches, including strict allergen avoidance and emergency epinephrine, are reactive rather than preventive and do not address the molecular pathways that initiate or perpetuate allergic disease [2]. As the incidence of food allergies continues to rise, there is a pressing need for model systems capable of dissecting the innate immune components that shape early responses to dietary allergens. Traditional mammalian models, especially mice, have provided insights into IgE-mediated hypersensitivity. Nevertheless, their use is limited by experimental constraints associated with oral sensitization, inhalation sensitization, and adaptive immunity, which can obscure the analysis of early innate signaling events [5,6]. *D. melanogaster* is particularly well suited to innate immunity investigation, due to the conserved nature of its immune pathways and lack of adaptive immunity [7,8]. Many human disease-related genes have clear homologs in Drosophila, and functional parallels between fly and mammalian immunity are well documented [9].

Innate immunity in *D. melanogaster* is mediated primarily by two Nuclear Factor-kappa B (NF-κB)-associated pathways: the Toll pathway and the Immune Deficiency (Imd) pathway. The Toll pathway is activated in response to fungi and Gram-positive bacteria and is structurally homologous to mammalian Toll-like receptor (TLR) signaling [9,10]. Upon activation, the inhibitory protein, Cactus (Cact), which is homologous to mammalian Inhibitor of kappa B (IκB), is degraded, allowing nuclear translocation of the NF-κB transcription factors, Dorsal (Dl) and Dorsal-related immunity factor (Dif), leading to the transcription of antimicrobial peptide (AMP) genes *drosomycin* (*Drs*) and *immune induced molecule 1 (IM1)* [11,12]. The Imd pathway responds mainly to Gram-negative bacteria and is homologous to mammalian Tumor Necrosis Factor (TNF) receptor signaling [13,14]. When activated, the NF-κB factor Relish (Rel) is proteolytically cleaved, allowing for transcription of AMP genes *Diptericin (Dpt)* and *AttacinA* (*AttA*) [12].

Emerging evidence suggests that allergens, much like microorganisms, can activate or modulate the innate immune pathways. A previous study demonstrated that *Dermatophagoides pteronyssinus* allergen 1 (Der p 1), the major allergen of the house dust mite, triggers activation of the Imd pathway via Rel and peptidoglycan recognition protein LC (PGRP-LC) [15]. This study established that allergens can act as immune stimuli in Drosophila. However, current research focuses largely on AMP induction rather than upstream regulators, such as Dl, Dif, Cact, or Rel, thus leaving gaps in our understanding of allergens’ influence on early innate immune signaling.

In mammals, peanut allergens are known to engage C-type lectin receptors (CLRs) and other innate components that lead to downstream inflammatory responses, thus suggesting the possibility that similar events occur in *D. melanogaster* [16]. Specifically, the degree to which TLRs play a role in driving the development of allergic responses to peanut remains unclear. Prior to this study, the interaction between food allergens and immune pathways in Drosophila had never been examined, specifically upstream immune regulators such as Dl, Dif, Cact, and Rel. Therefore, the present study aims to determine whether exposure to peanut induces measurable changes in innate immune gene expression in *D. melanogaster*. Because *D. melanogaster* lacks adaptive immunity, it cannot model a classical allergic reaction. Instead, it provides an opportunity to investigate how dietary allergens, such as peanut, may influence transcriptional changes in innate immune-associated genes and pathways.

## 2. Results

### 2.1. Survival Analysis

**Figure 1:**
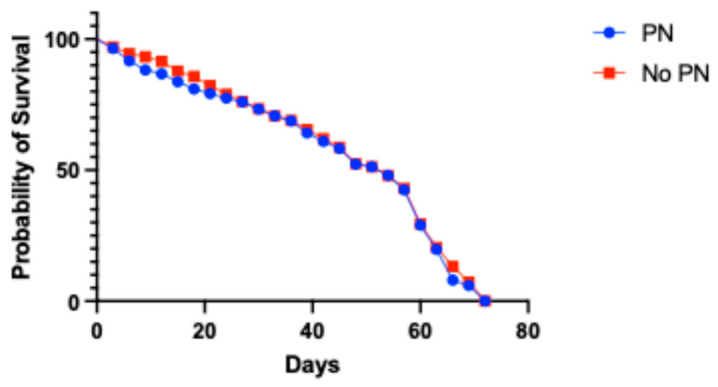
Survivorship analysis of PN-exposed flies and no PN controls. Kaplan–Meier survivorship analysis did not demonstrate a significant difference between the two groups (*p* = 0.4354).

The Kaplan–Meier survival analysis using a log-rank Mantel–Cox test showed no significant difference in the longevity between the peanut (PN)-exposed and no PN-exposed groups (*p* = 0.4354). The median survival for both groups was 54 days (Figure 1).

### 2.2. Gene Expression of PN-Exposed Versus No PN-Exposed Controls

**Figure 2:**
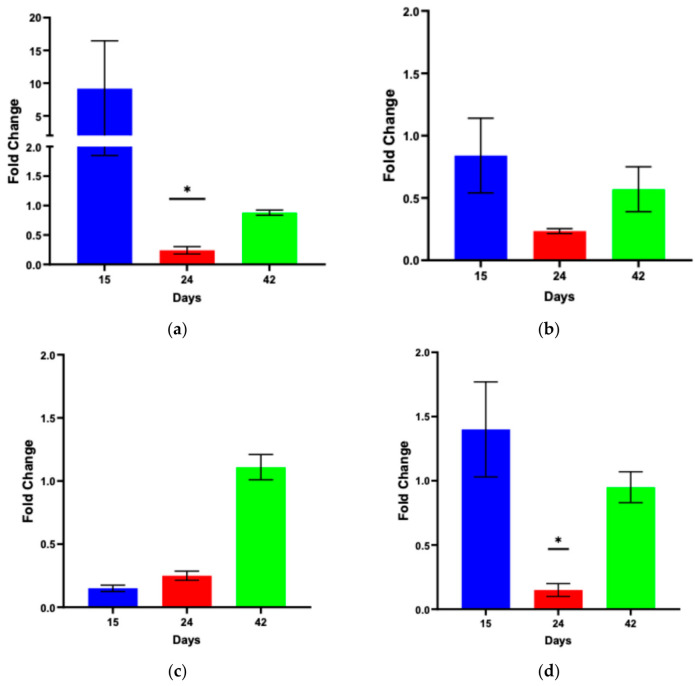
qRT-PCR analysis demonstrating average fold change in *Dl* (**a**), *Dif* (**b**), *Cact* (**c**), and *Rel* (**d**) gene expression in females at days 15, 24, and 42 between PN-exposed flies and no PN controls. Student’s *t*-tests determined that *Dl* and *Relish* are significantly downregulated at day 24 (*p* = 0.045; *p* = 0.016, respectively (marked by an asterisk)). The error bars represent standard error and n = 6.

Quantitative RT-PCR was used to determine relative gene expression at three time points (days 15, 24, and 42). Analysis of age-matched treatment and control groups revealed a significant downregulation of both *Dl* and *Rel* at day 24 (*p* = 0.045; *p* = 0.016, respectively; Figure 2). All other time points and gene expressions showed no significant differences (Figure 2).

**Figure 3:**
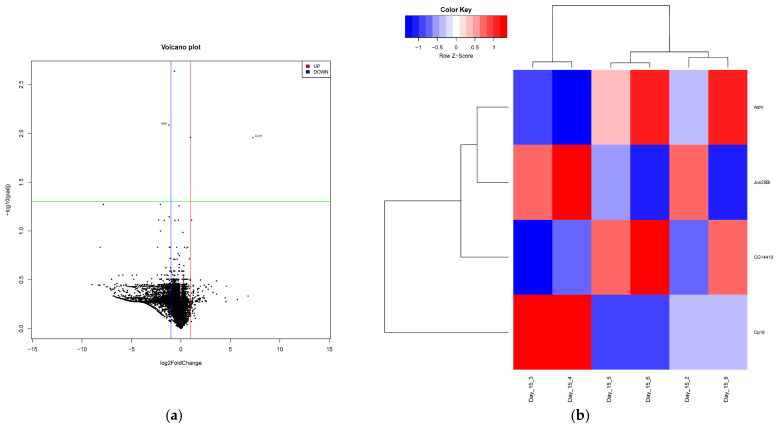
Differential gene expression in PN-exposed flies and no PN controls at day 15. (**a**) All genes are plotted on a volcano plot showing statistically significant up- (red) and downregulation (blue) (log_2_FoldChange above 1 (red line) or below −1 (blue line) and corrected *p*-value ≤ 0.05 (green line)). (**b**) 2D hierarchical clustering analysis of all significant genes (corrected *p*-value ≤ 0.05) showing statistically significant up- (red) and downregulation (blue).

**Figure 4:**
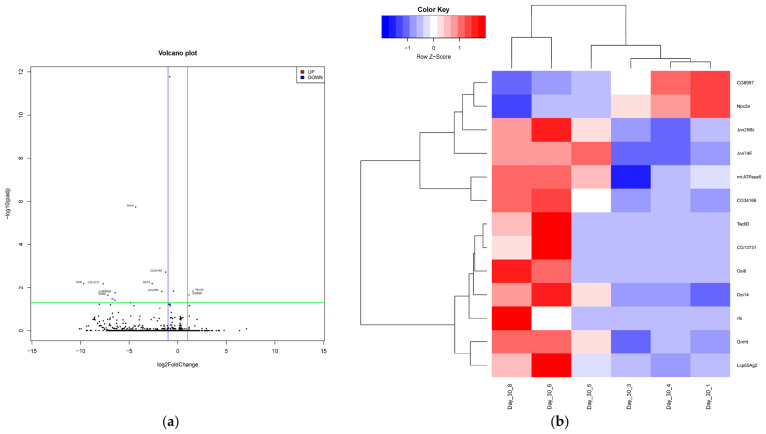
Differential gene expression in PN-exposed flies and no PN controls at day 30. (**a**) All genes are plotted on a volcano plot showing statistically significant up- (red) and downregulation (blue) (log_2_FoldChange above 1 (red line) or below −1 (blue line) and corrected *p*-value ≤ 0.05 (green line)). (**b**) 2D hierarchical clustering analysis of all significant genes (corrected *p*-value ≤ 0.05) showing statistically significant up-(red) and downregulation (blue).

Additionally, next-generation sequencing was conducted on PN-exposed and no PN-exposed total RNA extractions at days 0, 15, and 30 to evaluate the effects of PN exposure on whole-genome expression over time. Total RNA extractions for each time point were sequenced, and gene expression was evaluated by comparing the sequences to a reference *D. melanogaster* genome. Gene expression was measured by calculating fold change values between sets of samples and assessing statistical significance (*p* < 0.05). An additional cut-off for statistically significant genes was used to determine differentially expressed genes by evaluating fold change (log2 ≥ 1 or ≤ −1), categorized as either upregulated or downregulated. In the age-matched treatment and control groups, multiple genes were either up- or downregulated at day 15 and day 30, as shown by the volcano plot and heatmaps (Figure 3 and Figure 4). At day 15, *Cp18* (*chorion protein 18*) and *Jon25Bi* (*Jonah 25Bi*) were significantly upregulated (padj = 0.010982 and padj = 0.010982, respectively; Figure 3). Comparisons at day 15 also revealed that *CG14419* and *Adhr* (*Adh-related*) were downregulated (padj = 0.002302 and padj = 0.008221, respectively; Figure 4). At day 30, *Npc2e* (*Niemann–Pick type C-2e*) and *CG8997* were significantly upregulated (padj = 0.014671 and padj = 0.021739, respectively; Figure 4). Conversely, *mt:ATPase6* (*mitochondrial ATPase subunit 6*), *Jon74E* (*Jonah 74E*), *CG34166*, *Jon25Bii* (*Jonah 25 Bii*), *Osi14* (*Osiris 14*), *Gnmt* (*glycine N-methyltransferase*), *Lcp65Ag2*, *rib* (*ribbon*), *TwdlD* (*TweedleD), CG13731*, and *Osi6* (*Osiris 6*) were all significantly downregulated (padj = 0.014671, padj = 1.67 × 10^−12^, padj = 0.001968, padj = 0.015334, padj = 0.00657, padj = 1.83 × 10^−6^, padj = 0.017466, padj = 0.038688, padj = 0.022723, padj = 0.006766, and padj = 0.006535, respectively).

## 3. Discussion

This study provides novel evidence that *D. melanogaster* exposed to peanut exhibits measurable, yet modest, transcriptional changes in a limited number of genes. The first step in using this model was to confirm that PN exposure would not be lethal to the flies. As shown in Figure 1, the longevity and median survival of both treatment groups were not statistically significant. Further, each immune pathway required investigation at the transcriptional level: *Dl, Dif,* and *Cact* for Toll and *Rel* for Imd. Gene expression was assessed by qRT-PCR, and we found that at 24 days post-exposure, both *Dl* and *Relish* are significantly downregulated (Figure 2).

To better elucidate the interaction between PN exposure and immune activation, NGS was performed at 15 and 30 days post-exposure. In total, 17 genes were found to be differentially regulated: four at day 15 and 13 at day 30 (Figure 3 and Figure 4). Of the genes differentially regulated, 10 have known functions. Both *Cp18* and *Jon25Bi* were found to be upregulated at day 15 (Figure 3). *Cp18* is involved in chorion assembly and is a structural constituent of the chorion [17]. *Jonah* genes have shown exclusive expression in the Drosophila gut, leading to speculation that they are involved in the breakdown of dietary proteins, due to homology with mammalian trypsin and chymotrypsin [18]. However, other serine proteases have been implicated in the melanization reaction via proteolytic cleavage of prophenoloxidase [19]. Melanization is an important response in invertebrates triggered by microorganism entry and results in melanin deposition and phenol oxidation [20]. In contrast, two other *Jonah* genes (*Jon25Bii* and *Jon74E*) were found to be downregulated at day 30 (Figure 4), which was seen in a transcriptional response study using sigma virus to infect male and female flies [21]. Additionally, *Lcp65Ag2*, a structural constituent of the larval cuticle, and *ribbon* (*rib*) were also downregulated at day 30 (Figure 4). Several other genes were also found to be downregulated at day 30, including *mitochondrial ATPase subunit 6* (*mt:ATPase6*) and *TweedleD* (*TwdlD*; Figure 4). *mt:ATPase6* encodes a protein necessary for oxidative phosphorylation [22], and *TwdlD* encodes a chitin-binding protein and serves as a structural constituent of the larval cuticle [23]. Although these genes were differentially regulated, they are structural or metabolic genes. The difference in their expression may be due to the feeding, digestion, or processing of peanut rather than immune inactivation.

Also downregulated at day 30 (Figure 4) was *glycine N-methyltransferase* (*Gnmt*), but, in contrast to our findings, *Gnmt* has previously been shown to be upregulated during Toll activation. However, our findings could suggest a form of energy conservation, as the phenotype observed during that study was energy wasting due to lipolysis [24]. In mice, *Gnmt* downregulation or absence has been shown to increase inflammatory responses [25,26]. Additionally, flies overexpressing *Gnmt* exhibit increased longevity [27], further suggesting a detrimental response to PN exposure, but once again, this could be due to feeding on peanut. Further investigation will be needed to determine if *Gnmt* plays a role in innate immune activation.

At day 30, only one gene was significantly upregulated with a known function, *Niemann-Pick type C-2e* (*Npc2e*; Figure 4). This gene controls sterol homeostasis and steroid biosynthesis and has been extensively studied for its role in neurodegeneration. In humans, Niemann–Pick type C disease is a progressive neurodegenerative disorder marked by the accumulation of free cholesterol in late endosome- and lysosome-like compartments and results from a mutation in either *NPC1* or *NPC2*, in which *Npc2e* is a homolog for the latter [28,29]. More recently, *Npc2e* has been implicated in immune function within *D. melanogaster*. In flies challenged with toxic fungus *Aspergillus nidulans*, *Npc2e* was found to be significantly upregulated [30]. Similarly, during bacterial challenge with both Gram-positive and Gram-negative cells, multiple *npc2* genes were found to be upregulated, and NPC2 proteins were able to bind to lipopolysaccharide, peptidoglycan, and lipoteichoic acid, all components of the bacterial cell wall. Additionally, overexpression of NPC2e activated the *diptericin* promoter, an AMP produced via the Imd pathway, but not the *Drs* promoter, an AMP produced via the Toll pathway, in S2 cells [31]. While this observation is interesting in the context of immune reactions, we are cautious in the interpretation. Future studies will need to be performed to determine if this result is consistently observed.

Several limitations of this study should be acknowledged. First, changes in mRNA levels alone are not sufficient to demonstrate pathway activation. Future studies will need to include testing downstream canonical targets, reporter assays, and possibly pathway mutants/RNAi experiments. Secondly, whole-body RNA sequencing limits tissue-specific responses. Peanut is delivered through the diet; therefore, the gut should be examined, as well as the fat body for systematic immune responses. Finally, feeding rate and food preference were not controlled for; therefore, the metabolic gene changes could reflect altered feeding or nutrient intake. This also needs to be determined.

*In toto*, the results of this study indicate that peanut exposure is associated with limited transcriptional responses in *D. melanogaster*. This study provides an initial exploratory framework for examining how dietary allergens may influence immune-associated gene expression. Future studies will be required to determine whether these transcriptional changes reflect biologically meaningful immune responses.

## 4. Materials and Methods

### 4.1. D. melanogaster Husbandry and Longevity Experiment

Canton S wild-type *D. melanogaster* (Bloomington Drosophila Stock Center, Bloomington, IN, USA) were maintained in an incubator at 25 °C on a standard cornmeal, torula yeast, molasses medium with a diurnal light cycle. Once adequate stocks were established, stock bottles were expanded for fly collection by transferring into new bottles. The next generation of flies was allowed to eclose, and virgin female flies were collected for further analysis. Female flies were used based on previous mouse model studies showing that female mice undergo more severe anaphylaxis symptoms than their male counterparts [32]. The flies were placed in pint cages with air ventilation, a food vial, and an access point for a mouth aspirator to remove and add flies. The following conditions were established: peanut-exposed (PN), where food was supplemented with a 5% peanut solution allowed to dry on top, or no PN exposure, where distilled water was allowed to dry on top of the food. One hundred virgin female flies were added to an individual cage, and four cages were set up per condition, for a total of eight cages. The food was changed every 72 h, at which time dead flies were collected and stored at −80 °C for subsequent analysis. This was continued until all flies were dead (72 days). The statistical significance of longevity between treatment groups was determined using Kaplan–Meier analysis.

### 4.2. RNA Extraction and qRT-PCR Analysis

Total RNA extraction was performed at 15, 24, and 42 days post-exposure using TRIzol^®^ per the manufacturer’s instructions (ThermoFisher Scientific, Waltham, MA, USA). These sample dates were chosen as a preliminary method to determine if an immune response was induced when exposed to PN prior to the median survival (54 days). Each sample was quantified using a NanoDrop^TM^ ONE spectrophotometer (ThermoFisher Scientific) to assess RNA purity (260/280 ≈ 2.0) and concentration. TaqMan Gene Expression Assay kits (Applied Biosystems, Foster City, CA, USA) and a QuantStudio 5 Real-Time PCR System (Applied Biosystems) were used to perform reverse transcription quantitative PCR (qRT-PCR) according to the manufacturer’s instructions. Each reaction was prepared with 200 ng of RNA. The Taqman probe sets were *Ribosomal protein L32* (*RpL32*; endogenous control; assay #Dm02151827_g1), *Dorsal* (*Dl*; assay #Dm0180803_g1), *Dorsal-related immunity factor* (*Dif*; assay #DM01810798_g1), *Cactus* (*Cact*; assay #Dm0187757_g1), and *Relish* (*Rel* assay #Dm02134843_g1). Duplicate reactions for each of 3 experiments (n = 6) were carried out under the following conditions: 45 °C for 10 min and 95 °C for 10 min (95 °C for 15 s, 60 °C for 45 s) repeated for 40 cycles. The PCR products were analyzed in the linear range for amplification with RpL32 using QuantStudio 5 qPCR Data Analysis Software v1.5.1 (Applied Biosystems). Grubbs’ test was used to exclude any outliers within Ct values [33,34]. The relative quantitative results were used to determine changes in gene expression on a Log2 scale via the ΔΔCt method [35]. Ct values standardized to the reference gene *RpL32* (ΔCt values) were subjected to hypothesis testing with unpaired Student’s *t*-tests with a Welch correction, where appropriate [36].

### 4.3. Next-Generation Sequencing

An independent batch of virgin females was collected at days 0, 15, and 30 using the same method described above. Next-generation sequencing was used to compare triplicate samples of PN-exposed and no PN-exposed flies at different time points (0, 15, and 30 days post-exposure) to assess genes upregulated and downregulated during PN exposure. These days were chosen based on the preliminary results obtained from qRT-PCR. Specifically, days 15 and 30 were chosen to capture both early and late transcriptional changes. Day 30 was intended to capture sustained or downstream responses following the qRT-PCR time point (day 24) when the initial changes in *Dl* and *Rel* expression were detected. Total RNA samples were submitted to the UNMC Genomics Core Facility for quality control (QC), library preparation, and sequencing. Initial QC utilized the RNA integrity number (RIN), calculated from an Agilent 2100 Bioanalyzer, followed by mRNA-seq library preparation according to the Tecan (formerly NuGEN) Universal Plus mRNA-seq Library Preparation Kit protocol. Briefly, polyadenylated RNA was enriched from total RNA, followed by fragmentation, first and second strand cDNA synthesis, end repair, barcoded adapter ligation, and PCR amplification. Library quality and fragment size distribution were evaluated using a Bioanalyzer, and barcoded sample libraries were quantified and pooled prior to sequencing. Single-end 75 bp sequencing was performed on an Illumina NextSeq 550 platform using a High Output flow cell.

### 4.4. RNA Sequencing (RNA-Seq) and Bioinformatics Data Analysis

The original RNA-seq reads were trimmed by Trim Galore (Trim Galore! v0.6.7; available at https://github.com/FelixKrueger/TrimGalore; accessed on 13 May 2024) to remove adapters, terminal unknown bases (Ns) and low quality 3’ regions (Phred score < 20). The trimmed fastq files were processed by FastQC (FastQC: a quality control tool for high-throughput sequence data; available online at https://www.bioinformatics.babraham.ac.uk/projects/fastqc/; accessed on 13 May 2024) and MultiQC [37] for quality control. The trimmed fastq files were mapped to the BDGP6 Drosophila melanogaster reference genome (Ensemble 112 version) by the STAR aligner and were then subject to the RSEM tool for gene-level annotation and quantification [38,39]. The raw read counts were used for differentially expressed gene (DEG) analysis by the R package DESeq2 v1.44.0 [40]. The raw *p*-values were adjusted for the false discovery rate [41]. Significant DEGs were determined by padj ≤ 0.05. A volcano plot for each comparison was generated by R 4.3.1, and a 2D hierarchical clustering heatmap for each comparison was plotted by the gplots 3.1.3 package in R 4.3.1.

## Data Availability

The raw data supporting the conclusions of this article will be made available by the authors on request.
